# Supplementary material for: The TB vaccine clinical trial centre directory: An inventory of clinical trial centres in Sub-Saharan Africa
Source: PLoS One. 2024 Oct 28;19(10):e0292981. doi: 10.1371/journal.pone.0292981 (PMC11515998; doi:10.1371/journal.pone.0292981)
Supplement: S1 File — (PDF) [file pone.0292981.s001.pdf]

TB vaccine clinical trial site directory

The TuBerculosis Vaccine Initiative (TBVI), in collaboration with KNCV Tuberculosis foundation, is conducting an EDCTP-funded project with the aim to establish a directory of clinical trial sites suitable for future TB vaccine studies. The TB vaccine clinical trial site directory will help stakeholders with site selection for trials of future TB vaccine candidates and will be accessible on the EDCTP website. We will not include any personal information in the directory.

The aim of this survey is to develop a directory of clinical trial sites in sub-Saharan Africa and their capabilities to perform clinical trials for TB vaccine candidates. The survey contains 40 questions, and takes approximately 20-30 minutes to complete.

This survey is intended for one clinical trial site, in case there is multiple we kindly ask you to fill in multiple forms.

If information is unknown, please leave the answer blank. If an answer is not captured in the provided options, please enter the information in the "other" field.

For any further questions please contact info@tbvi.eu

* Required

1. Clinical trial site name *

2. Survey completed by (name) *

3. Date survey completed *

Please input date (M/d/yyyy)

4. Email-address *

5. Country *

6. City/ location *

7. What type of health facilities does the clinical trial site include?

- [ ] Academic institution
- [ ] Clinic
- [ ] Clinical research site
- [ ] District hospital
- [ ] Health center
- [ ] Laboratory
- [ ] National Hospital
- [ ] Regional Hospital
- [ ] University Hospital
- [ ] Public Hospital
- [ ] Primary Care Center
- [ ] Research group
- [ ] Other

8. Is the clinical trial site a stand alone site or affiliated with multiple clinical trial sites? *

- [ ] Single site
- [ ] Multiple sites
- [ ] Other

9. If multiple sites, is this located at one or multiple locations? *

- ◯ One location
- ◯ Multiple locations
- ◯ Other

10. In what year was the clinical trial site established?

11. Is the clinical trial site public or private ?

- ☐ Public
- ☐ Private
- ☐ Other

12. Is the clinical trial site for profit or non-profit?

- ☐ Profit
- ☐ Non-profit
- ☐ Other

13. Is the clinical trial site government or non-government owned?

- [ ] Government
- [ ] Non-government
- [ ] Other

14. What are the principal funding sources of the trial site

- ○ Industry
- ○ Academic
- ○ Investigator Initiated
- ○ Other

15. Is this trial site European & Developing Countries Clinical Trials Partnership (EDCTP) supported

- ○ yes
- ○ no
- ○ Other

16. Working languages of the trial site

- [ ] English
- [ ] French
- [ ] Portuguese
- [ ] Arabic
- [ ] Spanish
- [ ] Other

17. What is the (approximate) number of completed clinical trials at the trial site?

18. What trial phases capacity does the clinical trial site have? (multiple possible)

- [ ] Preclinical
- [ ] Phase 1
- [ ] Phase 2a
- [ ] Phase 2b
- [ ] Phase 3
- [ ] Phase 4

19. What therapeutic area is the trial site focused on?

- ☐ Tuberculosis (TB)
- ☐ Cholera
- ☐ Covid-19
- ☐ Ebola
- ☐ Influenza
- ☐ Malaria
- ☐ Human Immunodeficiency Viruses (HIV)
- ☐ Other

20. For any of these diseases what kind of clinical trials were conducted (multiple possible)?

- ☐ Infant vaccines
- ☐ Vaccines for adults
- ☐ Vaccines for adolescents
- ☐ Diagnostics
- ☐ Treatment regimens
- ☐ Other

21. For previous TB trials what kind of clinical trials were conducted (multiple possible)?

- [ ] TB infant vaccines
- [ ] TB vaccines for adolescents
- [ ] TB vaccine for adults
- [ ] TB diagnostics
- [ ] TB treatment regimens
- [ ] Other

22. If the clinical trial site has experience with Tuberculosis vaccines, for which targets is there capacity?

- [ ] Pre-exposure vaccines
- [ ] Post-exposure vaccines
- [ ] Not applicable
- [ ] Other

23. If the trial site has experience with Tuberculosis vaccines, which target population?

- [ ] Prevention of infection (POI)
- [ ] Prevention of disease (POD)
- [ ] Prevention of recurrent disease (POR)
- [ ] Therapeutic
- [ ] Not applicable
- [ ] Other

24. What participant population (demographics) the can be recruited in this clinical trial site

- [ ] Newborns
- [ ] Infants
- [ ] Children
- [ ] Adolescents
- [ ] Adults
- [ ] Older adults
- [ ] Household and close contacts of TB patients
- [ ] HIV- infected persons
- [ ] Drug resistant TB patients
- [ ] Healthcare workers
- [ ] Other

25. Please check the box if there is trial site population data available on:

- [ ] Population size
- [ ] Emigration rate
- [ ] TB incidence (pulmonary)
- [ ] TB infection prevalence
- [ ] Population HIV prevalence
- [ ] HIV prevalence among incident TB patients
- [ ] Retention rate
- [ ] BCG scar prevalence
- [ ] Tuberculosis preventive treatment (TPT)
- [ ] Drug resistant TB patients
- [ ] Other

26. What is the research capacity of the clinical trial site?

- [ ] Clinical research laboratories
- [ ] Research oversight
- [ ] Trial operational management,
- [ ] Demographic Health Survey (DHS) site
- [ ] Data management
- [ ] Other

27. If DHS, how many times per year is site used

28. What clinical facilities does the trial site have

- [ ] Outpatient clinics
- [ ] Laboratory
- [ ] Community clinics
- [ ] Emergency clinic
- [ ] Inpatient clinic
- [ ] Other

29. Is there community engagement in place at the trial site?

- ○ yes
- ○ no
- ○ Other

30. If yes, what type of community engagement

31. Is there patient support in place at the trial site?

- ○ yes
- ○ no

32. If yes, what type of patient support

33. Is there side effect management in place at the trial site?

- yes
- no

34. If yes, what type of side effect management

35. What is the length of follow up capacity at the trial site

- <5 months
- 1 year
- 3 years
- 5 years
- 10 years or more
- Other

36. Is there data certification management in place at the trial site?

- yes
- no
- Other

37. Is there Certified Authorization Professional (CAP) certification in place at the trial site

- yes
- no
- Other

38. Which capabilities of clinical testing are there at the trial site (multiple possible)

- [ ] Tuberculin skin test (TST)/ Mantoux test
- [ ] TB Interferon gamma release assays (IGRA)
- [ ] Sputum smear microscopy
- [ ] Light-emitting diode (LED) sputum smear microscopy
- [ ] Chest X-ray (CXR)
- [ ] Gene-expert (GXP)
- [ ] Drug susceptability testing (DST)
- [ ] Whole genome sequencing
- [ ] Hematology
- [ ] Biochemistry
- [ ] Enzyme-linked immunosorbent assay ELISA
- [ ] Solid culture
- [ ] Liquid culture
- [ ] HIV testing
- [ ] Other

39. Is there Laboratory Quality Management System (LQMS) implemented at the trial site

- ◯ yes
- ◯ no
- ◯ Other

40. What is the biosafety level of the laboratory facility at the trial site

- ◯ Level 1
- ◯ Level 2
- ◯ Level 3
- ◯ Level 4
- ◯ Not applicable
- ◯ Other

41. Thank you for your participation, please let us know if you have any additional information you would like to share

This content is neither created nor endorsed by Microsoft. The data you submit will be sent to the form owner.

Microsoft Forms
