# Supplementary material for: The TB vaccine clinical trial centre directory: An inventory of clinical trial centres in Sub-Saharan Africa
Source: PLoS One. 2024 Oct 28;19(10):e0292981. doi: 10.1371/journal.pone.0292981 (PMC11515998; doi:10.1371/journal.pone.0292981)
Supplement: S1 Table — WHO TB incidence rates [17] | Worldbank population [18]. (DOCX) [file pone.0292981.s002.docx]

# Supplement 2 Overview of centres contacted, and centres responded per country by TB incidence, population size, and country size

^WHO TB incidence rates^ (13) ^| Worldbank population^(14)
