# Supplementary material for: The TB vaccine clinical trial centre directory: An inventory of clinical trial centres in Sub-Saharan Africa
Source: PLoS One. 2024 Oct 28;19(10):e0292981. doi: 10.1371/journal.pone.0292981 (PMC11515998; doi:10.1371/journal.pone.0292981)
Supplement: S2 Table — (DOCX) [file pone.0292981.s003.docx]

# Supplement 3 Overview of the centres’ cities and country as represented in the clinical directory

| Country | City |
| --- | --- |
| Burkina Faso | Nouna |
| Cameroon | Yaoundé |
| Eswatini | Manzini |
| Ethiopia | Addis Ababa |
| Ethiopia | Addis Ababa |
| Ghana | Kintampo |
| Guinea-Bissau | Bissau |
| Kenya | HomaBay |
| Kenya | Nairobi |
| Kenya | Kericho |
| Kenya | Eldoret |
| Kenya | Kisumu |
| Malawi | Blantyre |
| Mali | Bamako |
| Mozambique | Manhiça |
| Nigeria | Ibadan |
| Nigeria | Enugu |
| Nigeria | Abakaliki |
| Senegal | Saint-Louis |
| South Africa | Cape Town |
| South Africa | Johannesburg |
| South Africa | Buffalo City Metro |
| South Africa | Ekurhuleni |
| South Africa | Klerksdorp |
| South Africa | Johannesburg |
| South Africa | Pretoria |
| South Africa | Johannesburg |
| South Africa | Bloemfontein |
| South Africa | Cape Town |
| South Africa | Durban |
| South Africa | Cape Town |
| South Africa | Cape Town |
| South Africa | Cape Town |
| South Africa | George |
| South Africa | Cape Town |
| South Africa | Durban |
| South Africa | Cape Town |
| South Africa | Johannesburg |
| South Africa | Johannesburg |
| South Africa | Durban/location |
| South Africa | Johannesburg |
| South Africa | Paarl |
| South Africa | Pretoria |
| South Africa | Cape Town |
| South Africa | Bloemfontein |
| South Africa | Johannesburg, Gauteng Province |
| South Africa | Durban |
| South Africa | Cape Town |
| Tanzania | Moshi |
| Tanzania | Coastal region |
| Tanzania | Dar Er Salaam |
| Tanzania | Mbeya |
| Tanzania | Mwanza |
| Uganda | Kampala |
| Uganda | Kampala |
| Zambia | Lusaka |
