# Supplementary material for: The TB vaccine clinical trial centre directory: An inventory of clinical trial centres in Sub-Saharan Africa
Source: PLoS One. 2024 Oct 28;19(10):e0292981. doi: 10.1371/journal.pone.0292981 (PMC11515998; doi:10.1371/journal.pone.0292981)
Supplement: Attachment — Submitted filename: Response to Reviewers.docx [file pone.0292981.s004.docx]

15 January 2024

Dear editors of PLOS ONE,

Many thanks for agreeing to consider our appeal and revised manuscript. As indicated by Jianhong Zhou on the 2th December, the editors encouraged re-submission and committed to re-review.

We thank the Reviewers for their comments and suggestions. We have adapted our manuscript according to the Reviewer’s comments and prepared a point-to-point response to their comments.

The present results have not been previously published, the dataset used for this manuscript is publicly available on the EDCTP website <https://www.edctp.org/our-work/coordination-tb-vaccine-funded-research/directory-tb-vaccine-clinical-trial-sites-sub-saharan-africa/>

We believe that the changes have improved the manuscript and trust that our manuscript now merits publication in the PLOS ONE.

Changes to the current manuscript text compared to the last submission are done using track changes.

Yours sincerely,

Puck Pelzer

Corresponding author on behalf of all co-authors

1. Please ensure that your manuscript meets PLOS ONE's style requirements, including those for file naming. The PLOS ONE style templates can be found at

<https://journals.plos.org/plosone/s/file?id=wjVg/PLOSOne_formatting_sample_main_body.pdf> and<https://journals.plos.org/plosone/s/file?id=ba62/PLOSOne_formatting_sample_title_authors_affiliations.pdf>

Answer: The authors have reviewed PLOS ONE’s style requirements and adapted accordingly

2. Thank you for stating the following financial disclosure:

“The directory was commissioned and funded by the EDCTP2 programme supported by the European Union and Development of tools to support the coordination of EDCTP TB vaccine funded research.”

Please state what role the funders took in the study.  If the funders had no role, please state: "The funders had no role in study design, data collection and analysis, decision to publish, or preparation of the manuscript."

If this statement is not correct you must amend it as needed.

Please include this amended Role of Funder statement in your cover letter; we will change the online submission form on your behalf.

Revised text amended Role of Funder statement: *The Funder had no role in study design, data collection and analysis of the data. The Funder was involved in the writing – review and editing – of the manuscript.*

3. Thank you for stating the following in the Acknowledgments Section of your manuscript:

We note that you have provided funding information that is currently declared in your Funding Statement. However, funding information should not appear in the Acknowledgments section or other areas of your manuscript. We will only publish funding information present in the Funding Statement section of the online submission form.

Please remove any funding-related text from the manuscript and let us know how you would like to update your Funding Statement. Currently, your Funding Statement reads as follows:

Funded by the European & Developing Countries Clinical Trials Partnership (EDCTP2), supported by the European Union, under the contract “Development of tools and documents to support the coordination of EDCTP TB vaccine funded research.”

Please include your amended statements within your cover letter; we will change the online submission form on your behalf.

Revised text *“**The directory of TB vaccine clinical trial centres suitable for future TB vaccine studies in Sub-Saharan Africa was conducted by TBVI and KNCV Tuberculosis foundation. The following experts provided advice on essential parameters that were included in the survey: Suzanne Verver, Frank Cobelens, Richard White and Rebecca Harris. We thank Ieva Leimane for her help with survey design, Daniela Pereira for data visualization, Degu Dare, Jeremy Hill, and Max Meis for technical support, and Stephanie Borsboom for project management. Our assessment relied partly on work done by KNCV for Aeras. Last but not least, we wish to thank all contact points from the centres for providing information.”/*

*“The directory was commissioned and funded by the EDCTP2 programme supported by the European Union and Development of tools to support the coordination of EDCTP TB vaccine funded research.”*

4. We noted in your submission details that a portion of your manuscript may have been presented or published elsewhere. [The background data is publicly available on the EDCTP website

<https://www.edctp.org/our-work/coordination-tb-vaccine-funded-research/directory-tb-vaccine-clinical-trial-sites-sub-saharan-africa/>] Please clarify whether this publication was peer-reviewed and formally published. If this work was previously peer-reviewed and published, in the cover letter please provide the reason that this work does not constitute dual publication and should be included in the current manuscript.

Answer: The present results have not been previously published, the dataset used for this manuscript is publicly available on the EDCTP website <https://www.edctp.org/our-work/coordination-tb-vaccine-funded-research/directory-tb-vaccine-clinical-trial-sites-sub-saharan-africa/>

5. We note that you have stated that you will provide repository information for your data at acceptance. Should your manuscript be accepted for publication, we will hold it until you provide the relevant accession numbers or DOIs necessary to access your data. If you wish to make changes to your Data Availability statement, please describe these changes in your cover letter and we will update your Data Availability statement to reflect the information you provide.

Revised text: *The dataset used for this manuscript is publicly available on the EDCTP website* [*https://www.edctp.org/our-work/coordination-tb-vaccine-funded-research/directory-tb-vaccine-clinical-trial-sites-sub-saharan-africa/*](https://www.edctp.org/our-work/coordination-tb-vaccine-funded-research/directory-tb-vaccine-clinical-trial-sites-sub-saharan-africa/)

6. We note that Figures 1 and 4 in your submission contain [map/satellite] images which may be copyrighted. All PLOS content is published under the Creative Commons Attribution License (CC BY 4.0), which means that the manuscript, images, and Supporting Information files will be freely available online, and any third party is permitted to access, download, copy, distribute, and use these materials in any way, even commercially, with proper attribution. For these reasons, we cannot publish previously copyrighted maps or satellite images created using proprietary data, such as Google software (Google Maps, Street View, and Earth). For more information, see our copyright guidelines: <http://journals.plos.org/plosone/s/licenses-and-copyright>.

We require you to either (1) present written permission from the copyright holder to publish these figures specifically under the CC BY 4.0 license, or (2) remove the figures from your submission:

1. You may seek permission from the original copyright holder of Figures 1 and 4 to publish the content specifically under the CC BY 4.0 license.

We recommend that you contact the original copyright holder with the Content Permission Form (<http://journals.plos.org/plosone/s/file?id=7c09/content-permission-form.pdf>) and the following text:

“I request permission for the open-access journal PLOS ONE to publish XXX under the Creative Commons Attribution License (CCAL) CC BY 4.0 (<http://creativecommons.org/licenses/by/4.0/>). Please be aware that this license allows unrestricted use and distribution, even commercially, by third parties. Please reply and provide explicit written permission to publish XXX under a CC BY license and complete the attached form.”

Please upload the completed Content Permission Form or other proof of granted permissions as an "Other" file with your submission.

In the figure caption of the copyrighted figure, please include the following text: “Reprinted from [ref] under a CC BY license, with permission from [name of publisher], original copyright [original copyright year].”

Answer: The maps were generated on the Visme platform, for which we have a subscription and access to templates.  In the [Terms and Conditions of Use](https://url.avanan.click/v2/___https:/www.visme.co/terms_conditions/___.YXAzOmlhdmk6YTpvOjI4MDcyNGQ4OGQ0NDg2OTY0ZGE0MGRjMzI5MGJlMjNmOjY6ZmRjNjoyNGRlYTlmNTViMzhjYTAyYjNiODc0NjZiMjYyOGQ3MTNkYmI5NmIzNjVkNjFlZmY3NzY3NjU3OTBlZWM0YjRjOmg6VA) there are multiple references to copyright, none applies to us. We used the template of the African continent for the shape of the map and used publicly available data for the WHO incidence estimates for input, plus the number of sites surveyed per country. We did not use Google software such as google maps, street view or earth. As there is no copyright owner of these maps we generated, there is no need for a Content Permission Form.

2. If you are unable to obtain permission from the original copyright holder to publish these figures under the CC BY 4.0 license or if the copyright holder’s requirements are incompatible with the CC BY 4.0 license, please either i) remove the figure or ii) supply a replacement figure that complies with the CC BY 4.0 license. Please check copyright information on all replacement figures and update the figure caption with source information. If applicable, please specify in the figure caption text when a figure is similar but not identical to the original image and is therefore for illustrative purposes only.

The following resources for replacing copyrighted map figures may be helpful:

USGS National Map Viewer (public domain): <http://viewer.nationalmap.gov/viewer/>

The Gateway to Astronaut Photography of Earth (public domain): <http://eol.jsc.nasa.gov/sseop/clickmap/>

Maps at the CIA (public domain): <https://www.cia.gov/library/publications/the-world-factbook/index.html> and <https://www.cia.gov/library/publications/cia-maps-publications/index.html>

NASA Earth Observatory (public domain): <http://earthobservatory.nasa.gov/>

Landsat: <http://landsat.visibleearth.nasa.gov/>

USGS EROS (Earth Resources Observatory and Science (EROS) Center) (public domain): [http://eros.usgs.gov/#](http://eros.usgs.gov/)

Natural Earth (public domain): <http://www.naturalearthdata.com/>

7. We notice that your supplementary 2 table are included in the manuscript file. Please remove them and upload them with the file type 'Supporting Information'. Please ensure that each Supporting Information file has a legend listed in the manuscript after the references list.

Answer: We have added supplementary table 2 to supporting information

8. Please review your reference list to ensure that it is complete and correct. If you have cited papers that have been retracted, please include the rationale for doing so in the manuscript text, or remove these references and replace them with relevant current references. Any changes to the reference list should be mentioned in the rebuttal letter that accompanies your revised manuscript. If you need to cite a retracted article, indicate the article’s retracted status in the References list and also include a citation and full reference for the retraction notice.

Answer: We have reviewed the reference list

[Note: HTML markup is below. Please do not edit.]

Reviewers' comments:

Reviewer's Responses to Questions

**Comments to the Author**

1. Is the manuscript technically sound, and do the data support the conclusions?

The manuscript must describe a technically sound piece of scientific research with data that supports the conclusions. Experiments must have been conducted rigorously, with appropriate controls, replication, and sample sizes. The conclusions must be drawn appropriately based on the data presented.

Reviewer #1: Yes

2. Has the statistical analysis been performed appropriately and rigorously?

Reviewer #1: Yes

3. Have the authors made all data underlying the findings in their manuscript fully available?

The [PLOS Data policy](http://www.plosone.org/static/policies.action#sharing) requires authors to make all data underlying the findings described in their manuscript fully available without restriction, with rare exception (please refer to the Data Availability Statement in the manuscript PDF file). The data should be provided as part of the manuscript or its supporting information, or deposited to a public repository. For example, in addition to summary statistics, the data points behind means, medians and variance measures should be available. If there are restrictions on publicly sharing data—e.g. participant privacy or use of data from a third party—those must be specified.

Reviewer #1: Yes

4. Is the manuscript presented in an intelligible fashion and written in standard English?

PLOS ONE does not copyedit accepted manuscripts, so the language in submitted articles must be clear, correct, and unambiguous. Any typographical or grammatical errors should be corrected at revision, so please note any specific errors here.

Reviewer #1: Yes

5. Review Comments to the Author

Please use the space provided to explain your answers to the questions above. You may also include additional comments for the author, including concerns about dual publication, research ethics, or publication ethics. (Please upload your review as an attachment if it exceeds 20,000 characters)

Reviewer #1: This is an important manuscript establishing a directory of clinical trial centers across sub-Saharan Africa to assess capacity for conducting late-stage TB vaccine trials. Considering this is the primary location of most future clinical trials this manuscript has important relevance and I recommend publishing. What I find important is the new information provided (available centers) but, perhaps just as valuable, also is locations where centers are not available and capacity is needed. I do have some comments to improve the flow and message of the manuscript.

How many of these sites are being used by the M72 trial?

Answer: The authors are unaware which sites have been selected for the M72 AS01 phase 3 trial.

Further discussion would be useful to discuss the dominance of South Africa in the findings. One sentence is given but I feel this is an important point and some further reasons should be added to the Discussion. Is there any collected information that could shed light on this? What are the implications of this?

Revised text*:* *There is over-representation of South African centres, which could be attributed to the country’s investments in TB vaccine centres*. *TB vaccine trials need to be conduced in areas with high TB incidence, which are typically in low-resource settings that lack capacity and prior trial experience. The marked prevalence of prior studies in South Africa positions it as an outlier in terms of capacity, elucidating the distinctive nature of its contribution to TB vaccine research. This concentration of research activity in South Africa emphasizes the needs for increased capacity needs for centres across Africa.*Top of Form
