# Supplementary material for: The TB vaccine clinical trial centre directory: An inventory of clinical trial centres in Sub-Saharan Africa
Source: PLoS One. 2024 Oct 28;19(10):e0292981. doi: 10.1371/journal.pone.0292981 (PMC11515998; doi:10.1371/journal.pone.0292981)
Supplement: Attachment — Submitted filename: Response to Reviewers_August24_final.docx [file pone.0292981.s006.docx]

1. It would be nice if: By summarizing the existing knowledge or previous efforts in mapping clinical trial centres for TB vaccine research in SSA i.e. a thorough literature review. This

would help highlight the importance of curating and maintaining an active directory for TB centres.

Answer: We thank the reviewer for their comment. Before the study began, a thorough desk search was conducted, and several experts were contacted to identify relevant information. To the authors' knowledge, no similar efforts have been undertaken in Sub-Saharan Africa, nor has such clinical trial centre map been curated elsewhere in the world. All available documentation on TB vaccine trial sites assessments were incorporated into the development of the directory. We hope this is stated adequately in the last paragraph of the introduction.

Revised text introduction section (line 169-175):

*Lessons learned from previous TB vaccine efficacy trials highlight the importance of innovative trial designs, selecting appropriate efficacy endpoints, and carefully choosing sites based on epidemiological data and target population. Performing such work requires access to clinical trial centres with adequate capacity, particularly for phase 3 licensure trials however, currently such information is not widely available. To address this gap, further advance TB vaccine development, and promote site selection, we curated a directory of clinical centres in SSA that are suitable for future TB vaccine studies, with possibility of regular updates.*

**Methods**

2. A copy of this 40-question online survey is missing in this submission (supplement 1) or a link to it is not given. Which survey is this? where does it originate from? was it adapted? how was it adapted to the SSA context? Providing details about the specific sections of this survey may help understand why the results are this way.

Answer: We apologize if the survey was not available to you with our previous submission. It is included in this revised version as supplement 1.

3. This methodology is not rigorous enough. The parameters although extensive in its coverage as a metric for the facilities, the desk review was not rigorous enough in its identification of clinical trial centres. Depending only on online data sources and excluding specific Ministry of Health Data across SSA countries that contain information of research-ready facilities and subsequent ward/county/or local governments. I suspect this is the reason South Africa comes out as dominant as most research-ready facilities have an online presence with prior work experience with clinical researchers from the global north as well as strong investments in Tb treatment. Focusing only on centres that have prior experience in Tb research does not bode well for regions with high incidence and low resources; Something other SSA countries might not have or formally documented.

Answer: We thank the reviewer for their feedback. We revised and reconstructed the method section (line 187-248). In the methodology section, we have clarified that the search was initiated by identifying centers with previous TB trial experience and after that, centers were identified more broadly (i.e. also without prior TB experience). The identification of centers was not a single online approach but included -beside the desk review-, the input from health ministers and it built on knowledge available through the EDCTP network and personal network of the experts. Additionally, the ClinicalTrials.gov, Centerfinder, Global Health Trials, Centrewatch, AAS trial centres, and AGT network were vetted for potential trial sites. We belief this triangulation of methods is sufficiently reliable within the means possible. We agree, that centers without formal documentation and/or low resources are potentially underrepresented in this directory. Therefore, we have added that as a limitation in the discussion and also emphasize the necessity of updating the directory regularly to accommodate for this.

Revised text method section (line 187-211):

*To identify trial centres and gather information on key parameters in SSA, a multistep approach was employed by triangulating and complementing information from both online resources, networks, and social media.*

*For the identification of the SSA centres, we triangulated information from a desk review and online sources using various websites, including ClinicalTrials.gov, Centerfinder, Global Health Trials, Centrewatch, AAS trial centres, and AGT network. We asked input from NTP managers, other experts, and made use of the EDCTP networks to identify additional centres and centres that potentially, did not have an online presence. Additionally in a second round of data collection, we used social media so that centres who were not yet identified but eligible for conducting TB vaccine trials could also be identified and reviewed for inclusion.*

*For the identification of essential parameters for the TB vaccine trials, a desk review was conducted which included a review of the existing literature on TB vaccine trials and reports. The resulting survey and directory parameters consisted of the centre characteristics, the type of disease targets and intervention, and expertise and the capacity (Table 1). A clinical trial centre directory template was developed along with data collection tools, including an Excel file and online survey. Data collection was carried out using a Microsoft Forms online survey tool, which contained 40 questions in English (S1 Survey). Four experts with knowledge on and/or experience with TB vaccine trials, reviewed the parameters, the data collection tool, and the survey design.*

Revised text in discussion section (line 368-371):

*The clinical trial centre assessment had some limitations. In the directory, centers without formal documentation and/or low resources are potentially underrepresented in this directory, of which the extent is unknown. However, we have tried to minimize the extent by complementing online sources with knowledge from experts and local NTPs.*

4. The paper could be improved by providing more details on the geographical distribution of the identified centres within their respective countries, beyond the over-representation of South African centres. I can guarantee that there are research-ready Tb centers in regions beyond the 10 centers identified in Nigeria (and the 3 that responded) as an example. This would help understand the regional coverage and potential gaps in the directory.

Answer: We thank the reviewer for their feedback. We have included a table with the countries and the cities represented in the clinical directory. Kindly see supplement 3 and the reference in General characteristics of the result section.

Revised text in result section (line 272-275):

*The majority of the 56 centres in the directory are located in South Africa (29), followed by Tanzania and Kenya with five centres each, and three in Nigeria. Two centres are in Uganda and Ethiopia, and the remaining nine countries have one centre each (Table 3 and see S3 Table for the centres’ cities).*

5.The authors mention the use of outdated epidemiological data on TB infection, which could limit the relevance of the directory. Discussing plans to incorporate more up-to-date epidemiological information would strengthen the utility of the directory.

Answer: We thank the reviewer for their feedback. We have removed that particular sentence in the discussion section as we did not collect epidemiologic data through the survey, we only asked if the data was available. Removed from line 393-394.

6. A final comment would be on the methods used to reach out to representatives of the centers identified. Most facilities under the administration of country MoH do not communicate directly with researchers; how did the authors navigate this without direct communication with country/county/state/regional MoH?

Answer: We thank the reviewer for their feedback. We contacted both the sites and the MoH representatives directly and neither gave us this feedback. If indeed this is the case, we consider this a limitation.

**Results**

7. The flow of information is somewhat confusing: Table 2 shows the clinical trial center capacity which is referenced in the text as table 3 in the second paragraph of the “General Characteristics section”. Furthermore, there seems to be no table 3 present in the submission.

Answer: We thank the reviewer for their feedback. There has been a mix up in the numbering of the tables. We have corrected this accordingly and changed the mentioning of table 3 into table 2 (see line 273).

8. I am curious as to why the supplement table includes data on Tb incidence vis a vis the number of facilities contacted and responded. The facilities contacted and responded could have easily been added to Table 2 to give a better indication of the research-ready facilities available that are responsive. Who is to say that a facility’s inability to respond means they are unable or not ready to handle vaccine trials? The Tb incidence data is something that should be alluded to in the introduction and to provide context as to why there is a need for responsive research-ready TB centers across SSA, additionally, this data is already provided in figure 4.

Answer: We thank the reviewer for their feedback. We agree with the reviewer that integration of table 2 and supplement table 2 would be more informative. We have therefore, added column “identified trial centers” and the “responded trial centers” to table 2. We also adapted our introduction to further highlight the relevance of understanding the capacity of trial sites for TB vaccine trials in SSA and mention the TB burden in the region as proposed. We have kept the data in figure 4 because we belief this visualization gives a quick impression of TB burden and sites available.

Revised introduction (line 103-108):

*Tuberculosis (TB) remains a significant global health threat, responsible for approximately 1.3 million deaths in 2020 alone (1), with the African region accounting for around a quarter of new TB cases worldwide. The burden of TB is especially acute in Sub-Saharan Africa (SSA). In this region, 16 of the 30 countries with the highest TB burden globally are located here. Despite a global reduction in TB incidence, SSA has seen a slower decline compared to the global average, highlighting an urgent need for targeted interventions.*

(line 119-125):

*In SSA, the intersection of TB and HIV presents a major public health challenge, as the region has one of the highest rates of HIV-TB co-infection worldwide (ref). HIV-positive individuals are at significantly higher risk of developing active TB, and their immune responses to vaccines can differ from those of HIV-negative individuals (ref). Furthermore, SSA is characterized by genetic diversity, both in human populations and in the strains of* Mycobacterium tuberculosis *circulating in the region. This diversity makes it essential to test new TB vaccines in SSA to ensure their effectiveness across different genetic backgrounds and pathogen strains.*

(line 137-153):

*Despite these advancements, the capacity for conducting TB vaccine trials remains limited globally, particularly in regions like SSA, which are most affected by the disease. Conducting TB vaccine clinical trials in SSA is important for several reasons. First, the high TB burden in the region provides the opportunity to test the efficacy of new TB vaccines in real-world settings where the disease is most prevalent. Second, SSA also has a high prevalence of HIV, which is a major risk factor for developing active TB. The high prevalence of HIV in SSA necessitates vaccine trials that can assess the performance of TB vaccines in this high-risk population. Third, the region's genetic and pathogen diversity requires vaccine efficacy to be evaluated across a wide range of backgrounds to ensure global applicability. Lastly, SSA's healthcare infrastructure, which often faces resource constraints, provides critical insights into the operational feasibility of vaccine deployment under real-world conditions.*

*Conducting TB vaccine trials in SSA promotes equity in global health research by ensuring that the populations most affected by the disease are not left behind in the development and rollout of new interventions. Mapping the clinical vaccine trial sites with capacity to conduct TB vaccine trials provides valuable insights in the current status of SSA to conduct trials, promotes site selection, and is essential for advocating for the expansion of trial capacity in SSA.*

9. The section could be strengthened by comparing the findings to other studies or directories of clinical trial centres. This would help to provide a more comprehensive understanding of the landscape of TB vaccine clinical trials in SSA.

Answer: We thank the reviewer for their feedback. We have included additional resources to relate this directory to however, this directory is the first of its kind. The result section is strictly meant for the description of the results and therefore, we have included any remarks to previous research in the introduction and the discussion. Please kindly see the introduction section line 169-175 and the section in the discussion can be found under point 10 of this letter.

10. The section could be improved by discussing the implications of the findings for policy and practice, such as how the directory can be used to inform the development of TB vaccine trials and improve the coordination of research efforts in SSA.

Answer: We thank the reviewer for their feedback. We have included a paragraph on the implications of the tool in the discussion section:

Revised discussion (line 390-401)

*As the first directory of its kind, this comprehensive listing of clinical trial centres in Sub-Saharan Africa (SSA) offers critical insights that can significantly impact decision-making on resource allocation and site selection for TB vaccine trials. Policymakers, research funders, and global health organizations can utilize this directory to prioritize sites with the necessary capacity and relevant experience. Furthermore, the directory facilitates improved coordination of research efforts across the region by identifying gaps in capacity and promoting collaboration between trial centres. This can help streamline the selection of trial sites, minimize duplication of efforts, and ensure that research is conducted efficiently across multiple locations. Additionally, the directory highlights countries that may require further investment to meet the demands of large-scale phase 3 trials, thus strengthening the research infrastructure in SSA. Ultimately, this directory serves as a vital tool for building a more coordinated and robust TB vaccine trial network in SSA, accelerating progress towards the global goal of ending TB.*

11. The section could be improved by discussing the importance of collaboration and coordination among researchers, policymakers, and funders to accelerate the development of TB vaccines and improve the coordination of research efforts in SSA; I am not sure this research took into consideration how much country Ministries of Health have done in terms of TB treatments.

Answer: We thank the reviewer for their feedback. We mention the coordination and collaboration between stakeholders in relation to the directory in the paragraph above, which we believe addresses point 11 sufficiently. While we do not discuss the Ministry of Health's coordination for TB treatments as this falls outside the directory's scope, we acknowledge that the complexity of gathering this information required the need for collaboration and coordination among researchers, policymakers, and funders and it is essential in the acceleration of TB vaccine development and to streamline research efforts in SSA.

**General comments**

12. Manuscript text should be double-spaced

Answer: We thank the reviewer for their feedback. We have double spaced the manuscript text.

13. Include page numbers and line numbers in the manuscript file

Answer: We thank the reviewer for their feedback. We have included page numbers and line numbers to the manuscript.

14. Vancouver” style is the reference style 🡪 Stata was not cited in Vancouver style

and does not appear in the reference list

Answer: We thank the reviewer for their feedback. We have included the reference for Stata in our reference list.

15. Figure captions are to be inserted immediately after the first paragraph in which

the figure is cited. Figure files are uploaded separately

Answer: We thank the reviewer for their feedback. We have removed the figures from the manuscript and inserted them in a separate document.

16. Tables are inserted immediately after the first paragraph in which they are cited.

Answer: We thank the reviewer for their feedback. We have moved the tables accordingly.

17. A consistent font be used throughout

Answer: We thank the reviewer for their feedback. We have corrected this in the manuscript.
