# Supplementary material for: The TB vaccine clinical trial centre directory: An inventory of clinical trial centres in Sub-Saharan Africa
Source: PLoS One. 2024 Oct 28;19(10):e0292981. doi: 10.1371/journal.pone.0292981 (PMC11515998; doi:10.1371/journal.pone.0292981)
Supplement: Attachment — Submitted filename: Reviewer comments.pdf [file pone.0292981.s005.pdf]

I am attaching this review as an extra to go through the fine points to help improve this paper section to section. In this paper the authors aim to develop a comprehensive directory of clinical trial centers in SSA a sort of “yellow pages” of centers that can support the coordination of Tb research. The directory includes information on centre characteristics, type of disease targets, intervention expertise, and capacity for TB vaccine trials. The study involved a desk review of existing centres and online surveys to gather data from 134 centres, of which 43% responded.

Introduction

The introduction is clear in its objective of developing this directory of Tb clinical trial centers in SSA, with a rationale aiming to strengthen capacity for Tb vaccine trials, given the Tb burden across the continent. It would be nice if;

- By summarizing the existing knowledge or previous efforts in mapping clinical trial centres for TB vaccine research in SSA i.e. a thorough literature review. This would help highlight the importance of curating and maintaining an active directory for Tb centers.

Methods

The study used a multi-pronged approach to identify and collect data from clinical trial centres in sub-Saharan Africa (SSA), including a desk review, online surveys, and expert recommendations. The use of an online survey tool with 40 standardized questions helps ensure consistency in the data collected across the participating centres.

- A copy of this 40-question online survey is missing in this submission (supplement 1) or a link to it is not given. Which survey is this? where does it originate from? was it adapted? how was it adapted to the SSA context? Providing details about the specific sections of this survey may help understand why the results are this way.
- This methodology is not rigorous enough. The parameters although extensive in its coverage as a metric for the facilities, the desk review was not rigorous enough in its identification of clinical trial centers. Depending only on online data sources and excluding specific Ministry of Health Data across SSA countries that contain information of research-ready facilities and subsequent ward/county/or local governments. I suspect this is the reason South Africa comes out as dominant as most research-ready facilities have an online presence with prior work experience with clinical researchers from the global north as well as strong investments in Tb treatment. Focusing only on centers that have prior experience in Tb research does not bode well for regions with high incidence and low

resources; Something other SSA countries might not have or formally documented.

- The paper could be improved by providing more details on the geographical distribution of the identified centres within their respective countries, beyond the over-representation of South African centres. I can guarantee that there are research-ready Tb centers in regions beyond the 10 centers identified in Nigeria (and the 3 that respondend) as an example. This would help understand the regional coverage and potential gaps in the directory
- The authors mention the use of outdated epidemiological data on TB infection, which could limit the relevance of the directory. Discussing plans to incorporate more up-to-date epidemiological information would strengthen the utility of the directory.
- A final comment would be on the methods used to reach out to representatives of the centers identified. Most facilities under the administration of country MoH do not communicate directly with researchers; how did the authors navigate this without direct communication with country/county/state/regional MoH?

Results

I commend the results section in presenting graphical visual aids in understanding however;

- The flow of information is somewhat confusing: Table 2 shows the clinical trial center capacity which is referenced in the text as table 3 in the second paragraph of the "General Characteristics section". Furthermore, there seems to be no table 3 present in the submission
- I am curious as to why the supplement table includes data on Tb incidence vis a vis the number of facilities contacted and responded. The facilities contacted and responded could have easily been added to Table 2 to give a better indication of the research-ready facilities available that are responsive. Who is to say that a facility's inability to respond means they are unable or not ready to handle vaccine trials? The Tb incidence data is something that should be alluded to in the introduction and to provide context as to why there is a need for responsive research-ready Tb centers across SSA, additionally, this data is already provided in figure 4.
- The section could be strengthened by comparing the findings to other studies or directories of clinical trial centres. This would help to provide a more comprehensive understanding of the landscape of TB vaccine clinical trials in SSA

Discussion

Overall, the Discussion section provides a clear summary of the main findings and implications of the study; however, due to some issues highlighted in the results section I will not be making substantial comments here as I still envisage a gap, however;

- The section could be improved by discussing the implications of the findings for policy and practice, such as how the directory can be used to inform the development of TB vaccine trials and improve the coordination of research efforts in SSA
- The section could be improved by discussing the importance of collaboration and coordination among researchers, policymakers, and funders to accelerate the development of TB vaccines and improve the coordination of research efforts in SSA; I am not sure this research took into consideration how much country Ministries of Health have done in terms of Tb treatments.

General comments in the writing/formatting

- Manuscript text should be double-spaced
- Include page numbers and line numbers in the manuscript file.
- Vancouver" style is the reference style → Stata was not cited in Vancouver style and does not appear in the reference list
- Figure captions are to be inserted immediately after the first paragraph in which the figure is cited. Figure files are uploaded separately.
- Tables are inserted immediately after the first paragraph in which they are cited.
- A consistent font be used throughout
